# Organizing an in-class hackathon to correct PDF-to-text conversion errors of *Genomics & Informatics* 1.0

**DOI:** 10.5808/GI.2020.18.3.e33

**Published:** 2020-09-17

**Authors:** Sunho Kim, Royoung Kim, Hee-Jo Nam, Ryeo-Gyeong Kim, Enjin Ko, Han-Su Kim, Jihye Shin, Daeun Cho, Yurhee Jin, Soyeon Bae, Ye Won Jo, San Ah Jeong, Yena Kim, Seoyeon Ahn, Bomi Jang, Jiheyon Seong, Yujin Lee, Si Eun Seo, Yujin Kim, Ha-Jeong Kim, Hyeji Kim, Hye-Lynn Sung, Hyoyoung Lho, Jaywon Koo, Jion Chu, Juwon Lim, Youngju Kim, Kyungyeon Lee, Yuri Lim, Meongeun Kim, Seonjeong Hwang, Shinhye Han, Sohyeun Bae, Sua Kim, Suhyeon Yoo, Yeonjeong Seo, Yerim Shin, Yonsoo Kim, You-Jung Ko, Jihee Baek, Hyejin Hyun, Hyemin Choi, Ji-Hye Oh, Da-Young Kim, Hyun-Seok Park

**Affiliations:** 1 Bioinformatics & Natural Language Processing Laboratory, ELTEC College of Engineering, Ewha Womans University, Seoul 03760, Korea; 2 Center for Convergence Research of Advanced Technologies, Ewha Womans University, Seoul 03760, Korea

**Keywords:** biomedical text mining, corpus, text analytics

## Abstract

This paper describes a community effort to improve earlier versions of the full-text corpus of *Genomics & Informatics* by semi-automatically detecting and correcting PDF-to-text conversion errors and optical character recognition errors during the first hackathon of *Genomics & Informatics* Annotation Hackathon (GIAH) event. Extracting text from multi-column biomedical documents such as *Genomics & Informatics* is known to be notoriously difficult. The hackathon was piloted as part of a coding competition of the ELTEC College of Engineering at Ewha Womans University in order to enable researchers and students to create or annotate their own versions of the *Genomics & Informatics* corpus, to gain and create knowledge about corpus linguistics, and simultaneously to acquire tangible and transferable skills. The proposed projects during the hackathon harness an internal database containing different versions of the corpus and annotations.

**Availability:** Five representative versions of *G&I* corpus constructed during the hackathon are uploaded onto ‘*G&I Hackathon 2020*’ folder of GitHub (https://github.com/Ewha-Bio/Genomics-Informatics-Corpus).

## Introduction

For biomedical text mining, it is necessary to use a corpus, which refers to a large and structured set of texts that have been electronically stored and processed. The full text of *Genomics & Informatics (G&I)* has been archived since 2003 as PDF files [1], and the content of the journal is available immediately upon publication without an embargo period. Even though the full-text publications of recent volumes are available as XML files, only scanned images or PDF files are available for earlier versions of publications, necessitating the conversion of images into machine-encoded text.

Thus, to build an initial version of the *G&I* corpus 1.0, we wrote a simple Python-based web crawler to directly browse and download PDF files from the *G&I* archives; then, we converted the PDFs into plain text files using PDFMiner or other optical character recognition (OCR) tools [2]. In this way, a prototype version of the full text-corpus of *G&I* 1.0 was recently archived in the GitHub repository, in 2018 [3].

Unfortunately, earlier versions of the *G&I* corpus 1.0 are of poor quality, and the noise induced by these errors present thorny issues for downstream standard text analysis pipelines, including tokenization, sentence boundary detection, and part-of-speech (POS) tagging, that would be used to develop the next version of the *G&I* corpus. Consequently, it was impossible to directly employ the obtained results for subsequent tasks without costly manual editing.

It was necessary to obtain motivated volunteers. To address this problem, the first event of *Genomics & Informatics* Annotation Hackathon (GIAH) was organized at Ewha Womans University, Korea to join forces for biomedical text mining with the goal of improving *G&I*; a hackathon is typically an event in which computer programmers and others involved in software development collaborate intensively over a short period of time on software projects [4].

Accurately extracting texts from PDF files has been an important issue for decades in the area of natural language processing and text mining. Nonetheless, we still do not have a definitive solution. In that sense, this hackathon tackled an important and not-yet-solved problem. Thus, our aim in the present paper is to describe a community effort to construct enhanced versions of the *G&I* corpus, in a consistent machine-readable format. We describe and summarize a collection of corpus projects reflecting achievements from this hackathon.

## Patterns of PDF-to-Text Conversion Errors

ASCII text and HTML text are human-readable formats. Text often comes in human unreadable formats, such as PDF files, that can only be opened using specialized software. Third-party libraries such as Adobe Acrobat Reader or PDFMiner provide access to these formats [2]. However, PDF conversion tools and OCR tools are still imperfect, as they occasionally misrecognize letters and falsely identify text, leading to misspellings and linguistic errors in the output text.

Most OCR conversion errors occur at line boundaries, where words are divided at the nearest break point between syllables, and a hyphen is inserted to indicate that the letters form a word fragment, rather than a full word. Thus, a word can be incorrectly separated (e.g., “se-parated” vs. “separated”). Many of these hyphenation errors could have been corrected, automatically, by applying some pattern-matching rules to these cases of hyphenation.

However, converting a PDF to a text file produces some odd and serious errors that need to be manually fixed. Thus, many errors need to be corrected manually, especially due to the fact that *G&I* contains many biomedical terms, many of which even contain special characters.

**Fig. 1.**
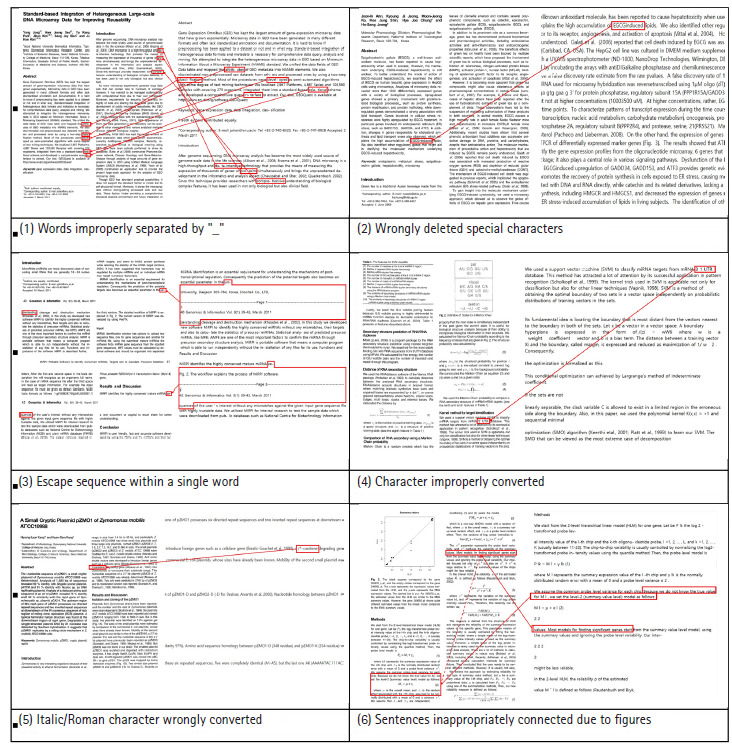
PDF to text conversion error types occurring in exemplary articles from *Genomics & Informatics (G&I)*.

Fig. 1 shows some of the exemplary patterns of errors that occur when converting a PDF file to text. A special character or hyphen can be omitted (e.g., *“miR26b”* vs. *“miR-26b”*; *“pvalue”* vs. *“p-value”*), or a character can be improperly converted into a different character (e.g., *“3' UTR”* vs. *“31 UTR”*). As to the problem of word boundaries, wrongly deleting white spaces (e.g., *“EGCGinduced”* vs. *“EGCG induced”; “2fold”* vs. *“2 fold”*), and wrongly inserting white spaces (e.g., *“differ- ences”* vs. *“differences”; “de- fined” *vs. *“defined”*) result in various incorrect split errors and run-on errors. Removing series of unnecessary white spaces is another problem (e.g., *“\r\r\n\r\nThe”* vs. *“The”*; *“http://www.sanger. ac. uk” *vs. *“http://www.sanger.ac.uk”*).

In many cases, non-word errors need manual correction, as they involve incorrect strings as well as misrecognized alphanumeric sequences with hyphenation (e.g., “TP53,” “protein-1,” “nuclear factor (NF)-kB,” “Benjamin-Hochberg,” “catechol-o-methyltransferase,” and “RT-PCR”).

## The First Event of the GIAH Hackathon and the Newly Built Corpora

The first event of the GIAH hackathon was held at the ELTEC College of Engineering of Ewha Womans University, 2020, with 76 participants, to enhance the *G&I* 1.0 corpus [4]. A meeting was held as a symposium to exchange and publicize the activities and ideas of improving the earlier volumes of the *G&I* corpus 1.0 (Vol. 1 to Vol. 9), explaining various issues and problems, as shown in Fig. 1. The participants worked on implementing their ideas with collaboration with other participants during a 2-week period.

Most of the teams initially applied regular expressions, correcting hyphenation, single-error misspellings, and a certain class of double-error misspellings, which are the major source of inaccuracies [5]. The corpus was processed and upgraded in several separate stages: manual editing by individuals, automatic editing by writing new pattern matching rules, and a checking and update loop to enhance the corpus, in an iterative cycle.

Various strategies were proposed based on composite machine-learning methods. Linguistic context-based error correction techniques were also used by most of the teams to detect and correct OCR errors with respect to their grammatical and semantic context [6-8]. Some participants proposed a method of automating the correction of misspelled words using on-line spell checkers [9]. This solution consists of using a lookup dictionary to search for misspelled words and correcting them suitably. Several teams used word embedding and deep learning techniques, such as Word2Vec, and BERT, with the idea of using context based on linguistic categories [10-14]. Still, this semi-automatic procedure is considered laborious and error-prone, as humans may miss some mistakes.

Many versions of the corpus were submitted. However, comparison of the performance of each project was difficult, as evaluation requires additional manual labor. Instead, we used several text comparison programs (open-source differencing and merging tools). These programs are highly useful for determining what has changed between different corpus versions, and then merging changes between versions.

**Fig. 2.**
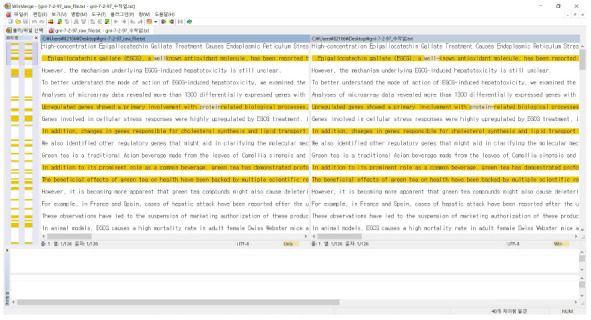
A screenshot of text comparison software (WinMerge) used to search for differences between two versions of texts (*G&I* 1.0 and the improved version) in order to highlight corrections made in *G&I* Vol. 7 No. 2 [15].

Fig. 2 shows a WinMerge [15] screenshot of error corrections, where a search was made for differences between two versions of texts (*G&I* 1.0 and the improved version) in order to highlight corrections made in *G&I* Vol. 7 No. 2. For example, 59 corrections were detected in the modified version of gni-7-2-97 (https://doi.org/10.5808/gi.2009.7.2.097) in the raw1 folder [16]. Among them, 30 were manual edits, and 29 were automatic edits. Likewise, 54 corrections were detected in the modified version of gni-7-2-111 file (https://doi.org/10.5808/gi.2009.7.2.111) in the raw1 folder [17]. Among them, 30 were manual edits, and 22 were automatic edits.

**Fig. 3.**
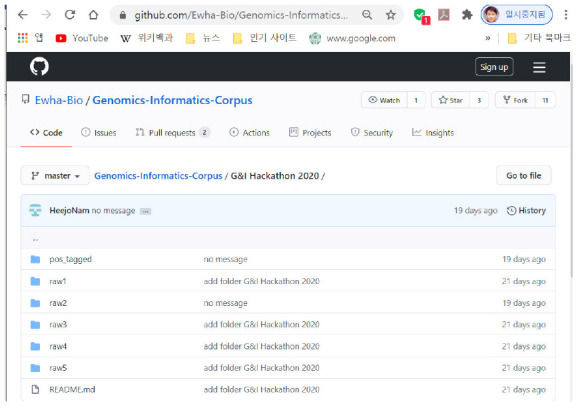
Five representative versions of the *Genomics & Informatics* corpus constructed during the hackathon are available through the subfolders of “*G&I Hackathon 2020*” of GitHub (https://github.com/Ewha-Bio/Genomics-Informatics-Corpus): raw1, raw2, raw3, raw4, and raw5.

Among all the submitted hackathon archives, the five best-performing versions of modified *G&I* corpus were selected and uploaded to subfolders of “*G&I Hackathon 2020*” on GitHub as shown in Fig. 3: *raw1, raw2, raw3, raw4*, and *raw5*.

Table 1 shows the number of files and updated lines in each of the five folders of GIAH hackathon archives. Among them, the raw1 folder (submitted by two participants, Sunho Kim and Royoung Kim) showed the best overall performance based on the number of manual corrections, the number of automatic corrections, documentation, and file coverage. We manually checked the error correction rate of randomly chosen files in the *raw1* folder, and on average, 30.3 occurrences of manual corrections and 24.1 occurrences of automatic corrections could have been detected per article, which are slightly larger numbers than were automatically detected by software in Table 1. Thus, the release of these improved corpora could potentially be a meaningful contribution.

## Conclusion

In this paper, we listed issues associated with upgrading the *G&I* corpus, and discussed methodological strategies to develop the next version of the *G&I* corpus based on a semi-automatic approach. Besides manual corrections, the outcome using pattern matching techniques and machine learning methods was noteworthy, and it greatly improved the error correction rate.

This is a progress report, and the current debate regarding our post-processing procedures focuses on how to ensure the quality of this semi-automatically modified corpus. It is taken as axiomatic that any correction must be confirmed by at least two, and usually more, people acting independently, so that their modification decisions can be compared. We suggest that a couple more rounds of the GIAH hackathon be organized to construct the future *G&I* 2.0 corpus. A semi-automatic method should be designed to build and improve the corpus, with a diminishing amount of manual checking.

## Figures and Tables

**Table 1.** Number of files and updated lines in five folders of the GIAH hackathon archives

	No. of files in the folder	No. of updated lines	Average No. of updated lines per file
raw1	183	8,513	46.5
raw2	*NA*	*NA*	*NA*
raw3	487	11,124	22.8
raw4	337	14,707	43.6
raw5	487	10,233	21

The statistics of the raw2 folder were unavailable for technical reasons. GIAH, *Genomics & Informatics* Annotation Hackathon.

## References

[1] Genomics and Informatics archives. Seoul: Korea Genome Organization, 2018. Accessed 2018 Jul 29. Available from: https://genominfo.org/articles/archive.php

[2] Shinyama Y PDFMiner.six: Python PDF parser and analyzer San Francisco: GitHub Inc., 2018. https://github.com/pdfminer/pdfminer.six.

[3] Oh SY, Kim JH, Kim SJ, Nam HJ, Park HS (2018). GNI Corpus Version 1.0: annotated full-text corpus of *Genomics & Informatics* to support biomedical information extraction. Genomics Inform.

[4] Briscoe G, Mulligan C (2014). Digital innovation: the hackathon phenomenon. Creativeworks London Working Paper No. 6.

[5] Kissos I, Dershowitz N OCR error correction using character correction and feature-based word classification.

[6] Mays E, Damerau FJ, Mercer RL (1994). Context based spelling correction. Inf Process Manag.

[7] Tong X, Evans DA A statistical approach to automatic OCR error correction in context.

[8] Foster J, Wagner J, van Genabith J Adapting a WSJ-trained parser to grammatically noisy text.

[9] Bassil Y, Alwani M OCR post-processing error correction algorithm using Google online spelling suggestion. https://arxiv.org/abs/1204.0191(2012).

[10] Mikolov T, Sutskever I, Chen K, Corrado G, Dean J Distributed representations of words and phrases and their compositionality.

[11] Mikolov T, Chen K, Corrado G, Dean J Efficient estimation of word representations in vector space. https://arxiv.org/abs/1301.3781.

[12] Peters ME, Neumann M, Iyyer M, Gardner M, Clark C, Lee K Deep contextualized word representations. https://arxiv.org/abs/1802.05365.

[13] Sharma A, Chaudhary DR (2013). Character recognition using neural network. Int J Eng Trends Technol.

[14] Garaas T, Xiao M, Pomplun M (2011). Personalized spell checking using neural networks. https://www.cs.umb.edu/~marc/pubs/garaas_xiao_pomplun_HCII2007.pdf.

[15] Varis K, Bradford D, Brimm D, Ganier L, Gerundt T, Rapp P WinMerge 2.14 Help. WinMerge. http://manuall.winmerge.org.

[16] Ahn JI, Jeong KJ, Ko MJ, Shin HJ, Chung HJ, Jeong HS (2009). High-concentration epigallocatechin gallate treatment causes endoplasmic reticulum stress-mediated cell death in HepG2 cells. Genomics Inform.

[17] Kim JM, Kim BG, Oh S (2009). Evolutionary signature of information transfer complexity in cellular membrane proteomes. Genomics Inform.

